# *Posidonia oceanica* (L.) Delile Dampens Cell Migration of Human Neuroblastoma Cells

**DOI:** 10.3390/md19100579

**Published:** 2021-10-15

**Authors:** Marzia Vasarri, Manuela Leri, Emanuela Barletta, Carlo Pretti, Donatella Degl’Innocenti

**Affiliations:** 1 Department of Experimental and Clinical Biomedical Sciences, University of Florence, Viale Morgagni 50, 50134 Florence, Italy; marzia.vasarri@unifi.it (M.V.); manuela.leri@unifi.it (M.L.); emanuela.barletta@unifi.it (E.B.); 2 Interuniversity Center of Marine Biology and Applied Ecology “G. Bacci” (CIBM), Viale N. Sauro 4, 57128 Livorno, Italy; pretti@cibm.it; 3 Department of Veterinary Sciences, University of Pisa, Viale delle Piagge 2, 56124 Pisa, Italy

**Keywords:** *Posidonia oceanica*, neuroblastoma, cell migration, autophagy, gelatinase, natural compounds

## Abstract

Neuroblastoma (NB) is a common cancer in childhood, and lethal in its high-risk form, primarily because of its high metastatic potential. Targeting cancer cell migration, and thus preventing metastasis formation, is the rationale for more effective cancer therapy against NB. Previous studies have described the leaf extract from *Posidonia oceanica* marine plant (POE) as an antioxidant, anti-inflammatory agent and inhibitor of cancer cell migration. This study aims to examine the POE anti-migratory role in human SH-SY5Y neuroblastoma cells and the underlying mechanisms of action. Wound healing and gelatin zymography assays showed that POE at early times inhibits cell migration and reduces pro-MMP-2 release into culture medium. By monitoring expression level of key autophagy markers by Western blot assay, a correlation between POE-induced cell migration inhibition and autophagy activation was demonstrated. Cell morphology and immunofluorescence analyses showed that POE induces neurite formation and neuronal differentiation at later times. These results suggest POE might act against cell migration by triggering early nontoxic autophagy. The POE-induced cellular morphological change toward cell differentiation might contribute to prolonging the phytocomplex anti-migratory effect to later times. Overall, these results encourage future in vivo studies to test POE applicability in neuroblastoma treatment.

## 1. Introduction

Neuroblastoma (NB) is the most common extracranial solid tumor of childhood accounting for 8–10% of all cancers in the pediatric population [1]. Even though the risk of many cancers in adults can be reduced with some lifestyle changes, there is currently no way to prevent most cancers in children because there are no NB-related environmental or lifestyle causes, and known risk factors, i.e., age and heredity, cannot be changed. 

NB presents with clinical behavior largely dependent on tumor biology [2]. Many cases of low-risk NB regress spontaneously or with conventional treatment options (5-year survival rate > 95%), but more than half of children with aggressive high-risk NB suffer refractory or relapsed disease with widespread metastases at diagnosis (5-year survival rate only about 40%) [3,4].

The foremost cause of cancer-related death involves metastasis, and the first step toward metastasis is the invasive capacity of cancer cells outside the primary tumor, driven primarily by their migratory capacity [5,6]. In high-risk NBs, tumor cells acquire the ability to degrade extracellular matrix (ECM) components to penetrate the basement membrane of blood vessels and metastasize by activating matrix metalloproteinases (MMPs) [7,8]. MMP-2 and MMP-9, also known as gelatinases, have been shown to play an important role in the invasion and metastasis of many cancers [9,10,11,12,13]. Therefore, targeting cancer cell migration, and thus preventing metastasis formation, is the rationale for more effective anticancer therapy [14,15].

In addition, NB arises from neural crest cell precursors of the sympathetic nervous system that fail to complete the differentiation process [16]. Repairing differentiation in NB could potentially arrest disease progression, as well as induce healthy mature neurons in patients by improving their life expectancy.

Furthermore, induction of cell differentiation has the advantage of reducing nonspecific toxicity compared to conventional therapies, as differentiating agents would theoretically have no effect on functional mature cells [17]. Retinoic acid (RA) is a known inducer of differentiation in NB cells [18,19]. Its 13-cis RA isoform is used in high-risk NB therapy [20], but with limited efficacy to treat minimal residual disease as maintenance therapy [21]; other RA isoforms with pro-differentiating, i.e., all trans retinoic acid (ATRA), and pro-apoptotic, i.e., 9-cis RA, activities have unfavorable pharmacokinetic and toxic profiles, limiting their use as NB treatments [17,22].

In light of this, identifying new agents able to act on multiple fronts, i.e., blocking cell migration and triggering a process of neuronal differentiation without leaving signs of cellular toxicity could be a winning weapon to manage the progression of high-risk NB by limiting the high toxicity associated with anticancer drugs.

For at least 40 years, the marine environment has been explored as a valuable resource of natural bioactive compounds potentially effective in various pharmacological applications against a variety of human diseases [23].

The marine plant *Posidonia oceanica* (L.) Delile is the only species belonging to the Posidoniaceae family endemic to the Mediterranean. The potential benefits of *P. oceanica* for human health start to times of yore. In ancient Egypt, *P. oceania* leaves were used as a herbal medication for skin issues and sore throats [24], for irritation and inflammation, and as a natural remedy for joint pain, acne and inflammation [25]. Additionally, the decoction of *P. oceanica* leaves was employed in the medical practice of western Anatolia inhabitants as a treatment for diabetes and hypertension [26]. These properties were scientifically proved by Goecke et al. in 2008 [26].

For years our group has been studying the bioactive properties of a hydroalcoholic extract from *P. oceanica* leaves (POE) [27]. A first UPLC characterization analysis showed that POE consisted of 88% phenolic compounds. Of these, about 85% was represented by (+) catechins, while the remaining 4% by a mixture of gallic acid, ferulic acid, epicatechin and chlorogenic acid. The small remaining fraction (11%) remained unknown/uncharacterized (Table 1) [10].

POE has been shown to have antioxidant, anti-inflammatory [28] and anti-glycation properties [29] as well as inhibitory role on cancer cell migration [10]. POE proved to be a cell-safe autophagy inducer [30], and a phytocomplex suitable for nanodelivery systems [31]. In an effort to contribute to the discovery of new natural products potentially useful for targeting NB cells, the current study explored the molecular mechanism underlying the anti-migratory activity of POE on SH-SY5Y cells, and its effects on cellular morphological change toward neuronal differentiation.

## 2. Results

### 2.1. Biochemical Properties and Antioxidant Activity of P. oceanica Leaf Extract (POE)

In this work, the hydroalcoholic extraction method previously described [10] allowed to recover 1.8 mg of dry extract per aliquot from 4 g of *P. oceanica* dried and minced leaves. Considering 0.5 mL of EtOH 70% (*v*/*v*) a standard resuspension volume, the concentration of hydrophilic analytes in a single batch was 3.6 mg/mL. 

POE was characterized for its total polyphenol (TP) and carbohydrate (TC) content using Folin-Ciocalteau and phenol-sulfuric assays, respectively. It was obtained that TP corresponded to 3.2 ± 0.1 mg/mL of gallic acid equivalents and TC to 6.3 ± 1.4 mg/mL of glucose equivalents (Table 2). POE hydrophilic compounds were also characterized for their antioxidant and radical scavenging properties; by colorimetric FRAP and DPPH assays, it was found that antioxidant and radical scavenging activity of POE corresponded to 1.2 ± 0.3 mg/mL and 10 ± 2.0 mg/mL, respectively, of ascorbic acid equivalents (Table 2).

These findings agree with those obtained from previous *P. oceanica* leaves extractions [28,29,30] supporting the efficacy and reproducibility of this hydroalcoholic extraction method.

### 2.2. POE Does Not Affect SH-SY5Y Cell Viability

**Figure 1:**
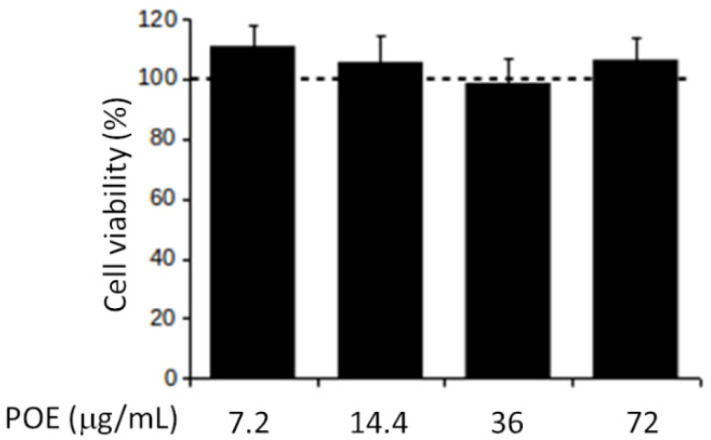
Effect of POE on cell viability. MTT assays on SH-SY5Y cells treated with different concentrations of POE for 24 h. Values are shown in percentage terms compared to vehicle-treated cells (dashed line). The amount of 70% (*v*/*v*) EtOH applied corresponds exactly to that in POE-treated cells (1:500, 1:250, 1:100 and 1:50 dilutions of 70% (*v*/*v*) EtOH). Values are plotted as mean ± SD of three independent experiments.

In this work, the effect of POE on cell viability was tested by MTT test at 24 h of treatment. Different dilutions of POE (1:500, 1:250, 1:100 and 1:50) corresponding to 7.2, 14.4, 36, to 72 μg/mL dry extract concentrations, respectively, were studied. Cells treated with 70% (*v*/*v*) EtOH vehicle were used as controls. As shown in Figure 1, POE did not lead to changes in cell viability, which remained comparable to that of control cells.

The lack of cytotoxicity confirmed the totally cell-safe profile of POE phytocomplex, so for the following assays POE was used at the lowest dose (7.2 μg/mL) in accordance with our previous works [10,28,30,31].

### 2.3. POE Blocks SH-SY5Y Cancer Cell Migration

The effect of POE on SH-SY5Y cell migration was evaluated by the wound healing assay. Cells treated with 70% (*v*/*v*) EtOH vehicle were used as controls (Ctrl). After scratching cell monolayer, the wound area was observed under a phase-contrast microscope and images were acquired at different times. The distance between wound edges was measured as an indicator of cell migration.

**Figure 2:**
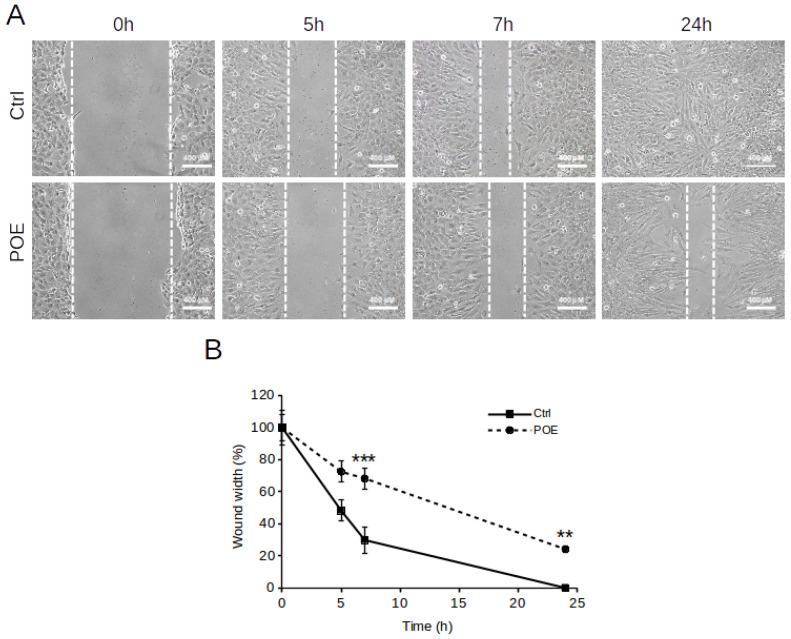
Effect of POE on SH-SY5Y cell migration. (**A**) Wound healing assay on SH-SY5Y cells treated with 70% (*v*/*v*) EtOH (Ctrl) or treated with POE (7.2 μg/mL). Scratch closure was monitored up to 24 h. Dashed lines mark the edges of the wound area. (**B**) Time course analysis of scratch closure in POE-treated cells or control cells. Wound width values were measured by considering the horizontal distance between the initial scratch and the scratch after migration at different time points. Values are represented as means ± SD from three different experiments. ** *p* < 0.01; *** *p* < 0.001 vs. Ctrl. Tukey’s test.

As shown in Figure 2A, POE caused a progressive slowing of cell migration already at a short time. Indeed at 5 h treatment, the wound was closed by approximately 30% (72 ± 6% wound width) in POE-treated cells compared to the initial scratch, whereas control cells migrated very rapidly reaching a 50% closure (48 ± 6% wound width) at 5 h from the initial scratch (Figure 2B). The inhibitory effect of POE on SH-SY5Y cell migration was sustained over time by maintaining 30% wound closure at 7 h of treatment (68 ± 6% wound width) and preventing complete closure at 24 h of treatment from the initial scratch (24 ± 2% wound width). In contrast, the highly migratory phenotype of control cells resulted in rapid wound closure of up to 70% (30 ± 8% wound width) at 7 h, and complete closure at 24 h from the initial scratch (Figure 2B).

These results agree with our previous insights into the anti-migratory ability of POE [10,30,31].

### 2.4. POE Prevents Gelatinase Activity

During cell migration process, ECM degradation is a key step that allows cells to migrate out of the primary tumor and metastasize to secondary areas. MMPs, in particular MMP-2/9 (or gelatinases), are involved in ECM degradation and therefore play an important role in cell migration process [7,8].

In this context, a potential effect of POE on gelatinase activity was examined by gelatin zymography assay. Since MMP-9 is generally secreted in negligible quantities by neuroblastoma cell lines, including SH-SY5Y cells [32], it was possible to observe and quantify the effect of POE mainly only on the activity of MMP-2 secreted in the culture medium.

**Figure 3:**
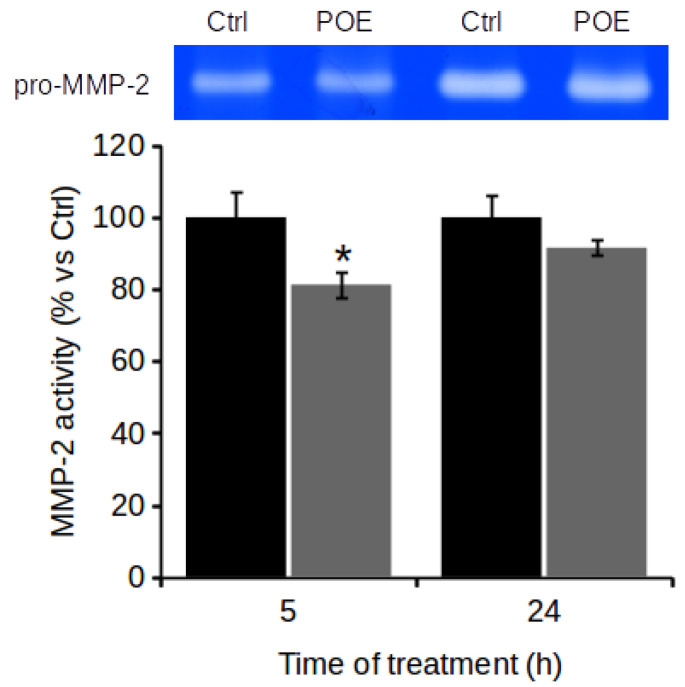
Effect of POE on MMP-2 release in culture medium. Gelatin zymography of cell culture media collected at 5 h and 24 h from SH-SY5Y cells cultured in absence or presence of POE (7.2 μg/mL). Data are reported as means ± SD from three different experiments. * *p* < 0.05 vs. Ctrl. Tukey’s test.

On the basis of the wound healing assay results, it was decided to investigate the MMP-2 activity in culture media collected after 5 h and 24 h POE treatment-time points at which POE showed anti-migratory activity. Cells treated with 70% (*v*/*v*) EtOH vehicle were used as controls (Ctrl). Gelatin zymography assay showed that SH-SY5Y cells with a migratory phenotype did not show active MMP-2 even under control condition. Instead, POE was found to cause a reduction in pro-MMP-2 activity of approximately 20% compared to control cells as early as 5 h of treatment (Figure 3). Even though minimal, this difference was statistically significant. Even though the amount of pro-MMP-2 released into the culture medium at 24 h was greater than that released at 5 h, irrespective of treatment, the inhibitory effect of POE on pro-MMP-2 activity tended to wear off at 24 h of treatment. At 24 h, pro-MMP-2 activity in POE-treated cells was comparable to that in control cells.

### 2.5. POE Triggers Autophagy Activation

Since several reports suggest that autophagy is involved in regulation of cell migration [33,34,35,36], we have also analyzed whether POE can affect cell migration by modulating autophagy activation. Therefore, activation of autophagy flux was examined over time during exposure of SH-SY5Y cells to POE. Cells treated with 70% (*v*/*v*) EtOH vehicle were used as controls (Ctrl).

**Figure 4:**
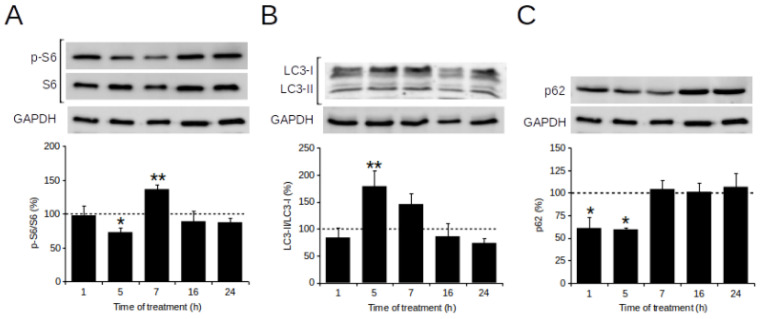
Effect of POE on autophagy activation. Representative images of Western blot analysis of (**A**) p-S6 and S6, (**B**) LC3 and (**C**) p62 in SH-SY5Y cells treated with POE (7.2 μg/mL). A densitometric analysis of three independent experiments was carried out to quantify signals intensity. Error bars represent standard errors. * *p* < 0.05; ** *p* < 0.01 vs. Ctrl (represented by dashed line); Kruskal–Wallis test.

In this work, the expression level of key autophagy markers in the mammalian target of rapamycin (mTOR) signaling pathway was monitored in POE-treated cells by Western blot analysis (Figure 4). Densitometric analysis of protein bands revealed that 5 h-POE treatment induced a significant 30% reduction in p-S6 (70 ± 7%), a downstream substrate of mTOR, compared with control cells (Figure 4A). At the same time, POE triggered a peak in the expression of LC3-II (179 ± 28%), the LC3-I lipid form indicative of autophagosome formation (Figure 4B). Finally, analysis of expression levels of p62, a marker of the degradative phase of autophagy, showed that 5 h-POE treatment caused a 40% (60 ± 2%) reduction in p62 levels compared with baseline levels in control cells (Figure 4C). 

The progressive increase in p-S6 status at 7 h-POE and the concomitant accumulation of p62 and reduction in LC3-II levels suggested that POE-induced transient autophagy activation was moving toward its final phase (Figure 4).

Representative Western blots images of the level of the three autophagic markers in control cells are shown in Figure S1 of “Supplementary Materials”.

**Figure 5:**
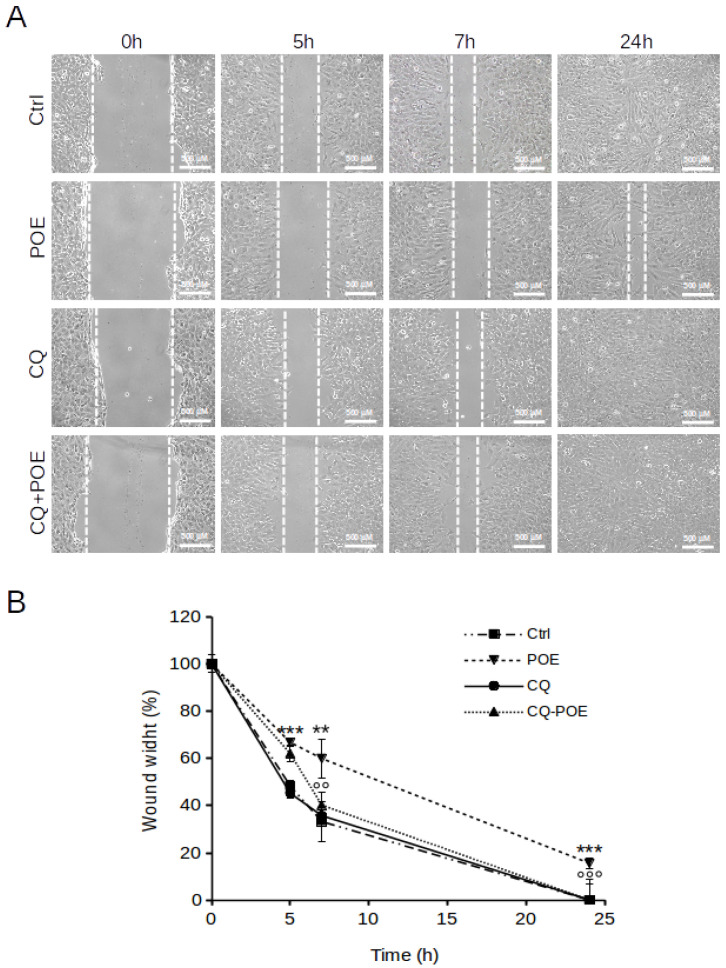
Evaluation of the regulatory role of POE-induced autophagy on SH-SY5Y cell migration by wound healing assay. (**A**) Representative image of SH-SY5Y cell migration after treatments with 70% (*v*/*v*) EtOH vehicle (Ctrl), POE (7.2 μg/mL), CQ (10 μM) and CQ-POE. Scratch closure was monitored up to 24 h. Dashed lines mark the edges of the wound area. (**B**) Time course analysis of scratch closure in SH-SY5Y cells treated with POE (7.2 μg/mL), CQ (10 μM) or CQ + POE, or vehicle-treated (Ctrl) in the wound healing assay. Wound width values were measured by considering the horizontal distance between the initial scratch and the scratch after migration at different time points. Data are representative of three different experiments. Error bars represent standard deviation. ** *p* < 0.01, *** *p* < 0.001 vs. Ctrl; °° *p* < 0.01, °°° *p* < 0.001 vs. POE-treated cells. Tukey’s test.

To further investigate the relationship between POE-induced autophagy and its effect on SH-SY5Y cell migration, chloroquine, which blocks acidification of lysosomes—the last phase of the autophagy process-, was added to POE-treated cells. Cell migration was observed by wound healing assay, and it was found that the presence of POE resulted in a stall in the kinetics of wound area closure starting at 5 h, in contrast to what was observed in control cells (Figure 5A). As depicted in the kinetic graph in Figure 5B, the wound was closed by approximately a 34% in POE-treated cells at 5 h (66 ± 2%) compared with the initial scratch, whereas control cells rapidly migrated to a 50% closure (48 ± 2%) compared with initial scratch, as discussed previously. After adding CQ at 5 h (time considered relevant for autophagy activation by POE) in POE-treated cells, it was obtained that at 7 h the wound was closed by approximately 60% (40 ± 6%) compared with the initial scratch, the cells had then resumed a highly migratory behavior similar to that of control cells (7 h-wound width of 33 ± 8%). Cells treated only with CQ were used as an additional control; their migratory behavior was similar to that of control cells. In contrast, cells treated with POE, without addition of CQ, migrated slowly while maintaining wound closure of 60% (59 ± 8%) at 7 h time point. At 24 h complete wound closure was observed in POE-treated cells added with CQ at 5 h, as well as control cells, whereas the presence of POE alone prevented complete wound closure while maintaining an opening of 15 ± 2%.

Taken together, these results support that POE-induced autophagy activation contributes to the inhibitory effect of the phytocomplex on SH-SY5Y cell migration. These data agree with our previous work on the role of POE against the highly migratory phenotype of human fibrosarcoma cells HT1080 [30].

### 2.6. POE Induces a Morphological Change towards Cell Differentiation

The literature reports that induction of cell differentiation can promote blockade of migration and invasiveness in human neuroblastoma cells [37]. In addition to the anti-migratory role of POE, this work examined whether the phytocomplex caused at later times a change on cell morphology and differentiation into mature neurons.

**Figure 6:**
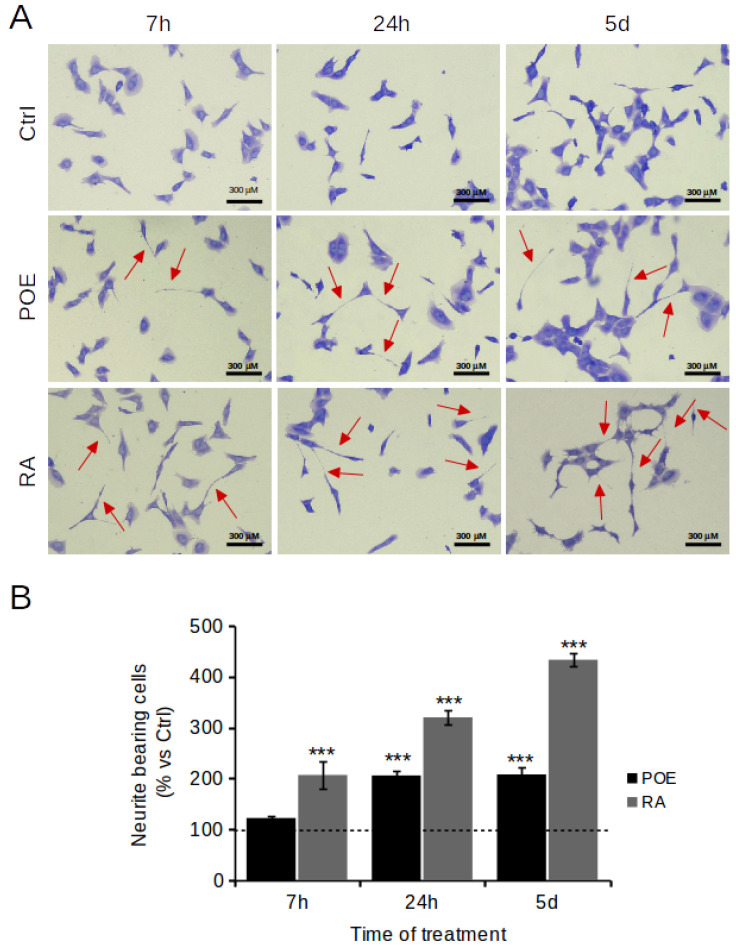
Effects of POE on neurite outgrowth in SH-SY5Y cells. (**A**) Cells were treated with POE (7.2 μg/mL) or RA (10 μM); vehicle-treated cells were used as control (Ctrl). Shown are images of cell fields stained with Coomassie Brilliant Blue R-250. (**B**) Neurite growth was quantified by counting the number of cells showing neurites that were twice as long as the cell body diameter in length. The proportion of cells with neurites was expressed as a percentage of the total number of cells. Approximately 200 cells were counted in each sample. Data are expressed as mean ± SD of three independent experiments. *** *p* < 0.001 vs. Ctrl.

Initially, the effect of POE on neurite outgrowth, a reliable feature of neuronal differentiation, was evaluated [38]. Neurite extension was measured in this study using a neurite outgrowth assay. After treatment with POE (7.2 μg/mL) in differentiating medium, SH-SY5Y cells showed distinct morphological changes consistent with neuronal differentiation, as evidenced by the development of long branching neurites (Figure 6A). The number of POE-treated cells bearing neurites was increased as early as 7h of treatment (122 ± 5%) to double at longer times—i.e., 5 days of treatment—(206 ± 5%) compared with untreated control cells (Figure 5B). The effect of POE on neurite outgrowth resembled that of retinoic acid (10 μM), although retinoic acid—a known and potent differentiating agent—induced up to 4-fold more neurites at 5-days treatment (433 ± 13%) compared with untreated control cells (Figure 6B).

**Figure 7:**
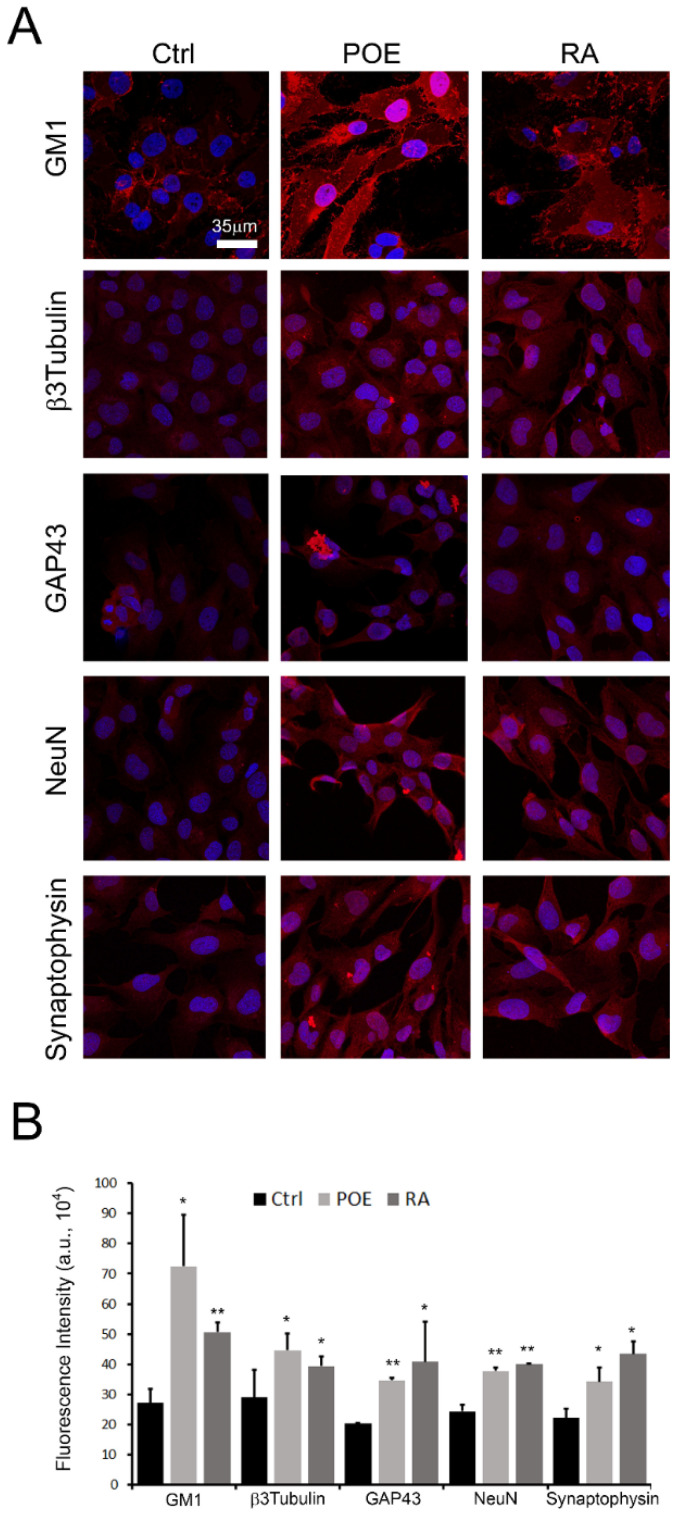
Effects of POE on expression of mature neuron markers in SH-SY5Y cells. (**A**) Immunofluorescence analysis by confocal microscopy of the membrane ganglioside GM1, β3Tubulin, GAP43, NeuN and Synaptophysin in SH-SY5Y cells treated with POE (7.2 μg/mL) or RA (10 μM) for 5 days; vehicle-treated cells were used as controls (Ctrl). Cells were stained with Alexa 488-conjugated CTX-B probe for GM1 detection, while with specific primary antibody and then with Alexa 568-conjugated anti-rabbit secondary antibody for detection of the other protein markers. (**B**) Quantification of red mean fluorescence intensity of almost 10 different acquisitions. One-way Anova Test, * *p* < 0.05; ** *p* < 0.01 vs. Ctrl; ** *p* < 0.01 vs. Syn treatment. Values are the average ± SE.

To verify the effect of POE on cell differentiation, the expression of several mature neuronal markers was analyzed by immunofluorescence analysis. As shown in Figure 7A, the expression levels of the membrane ganglioside GM1 (marker of neuronal differentiation and neuritogenesis), β3Tubulin (involved in the formation of neuronal processes), GAP43 (integral to growth cone formation, neuritogenesis and the development of a functional cerebral cortex), NeuN (nuclear protein expressed in most post-mitotic neurons of the central and peripheral nervous systems) and Synaptophysin (a critical component and marker for the presynaptic fusion complex) were analyzed [39,40,41]. From fluorescent signal quantification analysis, it was clear that 5-days POE cell treatment resulted in a significant increase in the expression of the above markers compared with control cells (Figure 7B). The expression of these markers in RA-treated cells for 5 days was used as a control of the cellular differentiation process.

In summary, these results collectively indicate that POE is able to trigger a morphological change toward neuronal differentiation.

## 3. Discussion

Tumor cell migration is a crucial step in tumor metastasis [42], which is the most important cause of neuroblastoma-related deaths [3,4]; therefore, targeting tumor metastasis is a promising approach for the treatment of neuroblastoma.

During the process of tumor invasion, gelatinases have long been recognized as primarily responsible for proteolytic degradation of the extracellular matrix. Inhibiting the expression and activity of MMPs is an active area of research in the prevention of tumor metastasis. In fact, the literature describes many flavonoids and polyphenols, along with saccharoids and fatty acids, as the most important groups of MMPs inhibitors derived from marine natural products [43]. In this work, wound healing assay clearly showed that POE phytocomplex, composed mainly of polyphenols, effectively inhibited SH-SY5Y cell motility. However, SH-SY5Y cells did not show active MMP-2 even under control conditions. This prevents us from correlating POE-induced migration inhibition with a reduction in active MMP-2. However, POE has been clearly shown to inhibit pro-MMP-2 expression. Therefore we can speculate that this effect of POE on the downregulation of pro-MMP-2 might reduce the in vivo invasiveness related to activation of pro-enzyme by activators present in the tumor microenvironment thus contributing to reduce the invasiveness of neuroblastoma. These results reinforce previously described data on the POE mechanism of action against cancer cell migration [10,30], and suggest that POE might play a role in cell motility and invasiveness by different molecular mechanisms underlying cell motility and cell invasiveness.

Furthermore, the literature reports that cell migration is a finely regulated process, and that autophagy may have a control over it [30,33,34,35,36]. In this work, the possible relationship between POE-induced inhibition of cell migration and autophagy activation was tested. The expression of key autophagy markers in POE-treated SH-SY5Y cells was monitored over time, and in particular the effect of POE on the autophagy mTOR-dependent pathway was investigated [44]. Western blot results showed that expression levels of the S6 phosphorylated form (p-S6), a downstream substrate of mTOR, underwent a transient and significant reduction in POE-treated cells. The resulting activation of autophagic flux was confirmed by the concomitant increase in the levels of LC3-II/LC3-I, an index of autophagosome formation and reduction in the levels of p62, a marker of the final step of autophagic degradation.

Our data show that the peak of autophagic activation at 5h POE treatment coincided temporally with the maximal POE inhibitory effect on SH-SY5Y cell migration. The resumption of the highly migratory phenotype of SH-SY5Y cells after addition of the autophagy inhibitor CQ, at 5 h POE treatment, confirmed that the activated autophagy played a key role on the regulation of cell migration.

In addition, the cell viability analysis by MTT assay confirmed that POE bioactivities occur without any signs of cytotoxicity, thus confirming the non-toxic potential of POE [10,28,30,31].

Our most interesting finding concerns the effect of POE on cell morphology. Coomassie Brilliant Blue R-250 staining showed that POE treatment had a clear effect on neurite formation. Since neurite extension is a reliable feature of neuronal differentiation [45], morphological changes toward cellular differentiation after POE treatment at longer timescales were investigated.

By immunofluorescence analysis, it was observed that POE resulted in the increase of some of the key markers of neuronal differentiation—i.e., GM1, β3Tubulin, GAP43, NeuN, Synaptophysin—compared to control cells, while maintaining a morphological profile similar to that of RA-treated cells.

Given this evidence, we might hypothesize that the 5-days POE-induced morphological change toward cell differentiation might contribute to the maintenance of POE-induced cell migration inhibition at longer times. The literature reports that treatment with retinoic acid, the most used inducer of cell differentiation, reduces cell migration and invasiveness in human neuroblastoma cells, blocking one of the main features underlying cancer progression [37].

To date, one of the therapeutic approaches for the high-risk NB is the differentiation therapy. Induction of neuroblastoma cell differentiation has the advantage of reducing nonspecific toxicity compared to conventional therapies, as differentiating agents theoretically do not affect functional mature cells [46]. Even though retinoic acid is the most effective differentiating agent available for the treatment of neuroblastoma, it is not without cellular toxicity. Therefore, the possibility of inducing differentiation of SH-SY5Y cells with a nontoxic natural agent-albeit with lower efficacy than retinoic acid-could open new perspectives for treatment in single or in combination with low-dose retinoic acid in order to reduce the toxicity of conventional therapy.

This work collectively provides background information on the role of POE against the highly migratory behavior of SHSY5Y cells. Inhibiting tumor cell migration by cell-safe modes of action would mean intervening on cancer progression in a safer manner.

In conclusion, this study provides new insights into the in vitro POE anti-migratory role in SH-SY5Y human neuroblastoma cells. POE was found to act on multiple targets at an early stage, i.e., it inhibits pro-MMP-2 expression and activates a cellular autophagy flux. The ability of POE to induce a morphological change toward cell differentiation in mature neurons at later times might promote long-term inhibition of cell migration.

These in vitro results need further rigorous studies to investigate the biological efficacy of POE in appropriate in vivo animal models in order to confirm and expose our results before considering POE as a possible anti-tumoral agent for the neuroblastoma management.

## 4. Materials and Methods

### 4.1. Materials and Reagents

Dulbecco’s Modified Eagle Medium (DMEM), Ham’s F-12 Nutrient Mixture, Fetal Bovine Serum (FBS), L-glutamine, penicillin and streptomycin, 3-(4,5-Dimethylthiazol-2-yl)-2,5-Diphenyltetrazolium Bromide (MTT), 2,2-diphenyl-1-picrylhydrazyl (DPPH), 3-(2-Pyridyl)-5,6-diphenyl-1,2,4-triazine-p,p′-disulfonic acid monosodium salt hydrate (Ferrozine^®^), Folin–Ciocalteau’s reagent, gallic acid, ascorbic acid, D-glucose, Coomassie Brilliant Blue R-250, gelatin and all other chemicals and solvents were purchased from Merck KGaA (Darmstadt, Germany). Electrophoresis reagents were purchased from Bio-Rad (Hercules, CA, USA). Disposable plastics were from Sarstedt (Nümbrecht, Germany).

The leaves of *P. oceanica* were collected in July 2020 in the protected marine area of Meloria by the authorized and qualified staff of the Interuniversity Center of Marine Biology and Applied Ecology “G. Bacci” (Leghorn, Italy) at a depth of about 15 m at the following geographical coordinates: 43°35′13″ N and 010°10′21″ E.

### 4.2. P. oceanica Extraction Method

Hydrophilic compounds of *P. oceanica* leaves were obtained as previously explained [10]. To summarize, dried and minced *P. oceanica* leaves were suspended overnight in 10 mL of an 70% (*v*/*v*) EtOH solution per gram of leaves at room temperature under stirring conditions, and then left for 3 h at 65 °C. The hydroalcoholic extract was split up from the debris by centrifugation at 2000× *g*; *n*-hexane was added to the supernatant in a 1:1 ratio. Hydrophobic compounds were removed after repeated agitation, while hydrophilic compounds were recovered in the lower phase of the extract.

Hydrophobic compounds were removed after repeated shaking, whereas hydrophilic compounds were recovered in the lower phase of the extract. The hydrophilic phase was then dispensed in 1 mL aliquots and dried with a Univapo^TM^ vacuum-spin concentrator. Prior to use, a single batch of *P. oceanica* extract was dissolved in 0.5 mL of 70% (*v*/*v*) EtOH and referred to as POE.

The total polyphenol and carbohydrate content of POE, as well as its antioxidant and radical scavenging activities, were evaluated according to previously described colorimetric methods [10,28].

### 4.3. Cell Line and Culture Conditions

American Type Culture Collection (ATCC^®^, Manassas, VA, USA) provided the human neuroblastoma SH-SY5Y cell line. Cells were cultured in a 1:1 mixture of Ham’s F12 and DMEM supplemented with 2 mM L-glutamine, 100 mg/mL streptomycin, 100 U/mL penicillin and 10% FBS (complete medium), in a humidified 5% CO2 incubator. At 90% confluence, SH-SY5Y cells were propagated by trypsinization (0.25% trypsin, 0.5 mM EDTA solution) detachment.

The experiments were performed in heat-inactivated serum medium (HI-FBS medium) in the absence or presence of POE. To test the effect of POE on cell differentiation, cells were seeded in complete medium with 3% reduced FBS (differentiating medium) in the presence of POE (7.2 μg/mL), added to the medium every day for five days. Cells treated with RA (10 μM) for 5 days in differentiating medium were used as a control for cell differentiation [41]. Cells treated with 70% (*v*/*v*) EtOH vehicle were used as controls (Ctrl).

### 4.4. Cell Viability Assay

MTT test was used to assess SH-SY5Y cell viability. Cells were seeded into 96-well plates at a density of 5 × 10^3^ cells/well overnight. Then, cells were treated with several POE dilutions corresponding to 7.2, 14.4, 36 and 72 μg/mL dried weight of extract for 24 h in HI-FBS medium.

Cells treated with 70% (*v*/*v*) EtOH were used as a control. After removing culture medium, 100 mL of MTT solution (0.5 mg/mL) was added to each well for 1 h at 37 °C in the dark. Next, cells were lysed with a lysis solution (20% SDS, 50% N,N-dimethylformamide). Absorbance values were measured at 595 nm using an iMARK microplate reader (Bio-Rad, Philadelphia, PA, USA).

Data on relative cell viability were expressed in terms of percentage of the untreated control cells.

### 4.5. Wound Healing Assay

Migration of SH-SY5Y cells was assessed by wound healing assay. Cells were seeded in 6-well plates at a density of 5 × 10^5^ cells/well. Upon reaching confluence, a longitudinal scratch was performed through the cell monolayer using a 200 μL sterile plastic tip. 

After a PBS washing for removing non-adherent cells, SH-SY5Y cells were treated with POE (7.2 μg/mL) in HI-FBS medium for up to 24 h. Untreated cells were used as a control.

Cell-free area was observed by phase-contrast microscopy, and images were captured at 0, 5, 7 and 24 h after wounding using a Nikon TS-100 microscope equipped with a digital acquisition system (Nikon Digital Sight DS Fi-1, Nikon, Minato-ku, Tokyo, Japan). Marked edges along each wound were used to measure cell migration by considering the horizontal distance between the initial scratch and the scratch following migration.

### 4.6. Gelatin Zymography

Gelatinase activity was assessed by gelatin zymography using as previously described [30]. Briefly, cells were seeded at a density of 2 × 10^5^ cells/well in 24-well plates and incubated overnight. Cells were then treated with POE (7.2 μg/mL) in HI-FBS medium for up to 24 h, while untreated cells were used as control.

Culture supernatants were collected and centrifuged at 9700× *g* for 1 min at 4 °C in order to pellet cellular debris. Then, aliquots of conditioned media (2.5 μL) were electrophoresed under non-reducing conditions in a 8% polyacrylamide gel containing gelatin (1 mg/mL).

The gel was washed twice (30 min/wash) in 2.5% (*v*/*v*) Triton X-100 for 1 h to facilitate SDS removal and then incubated at room temperature for 30 min in reaction buffer (50 mM Tris-HCl pH 7.4, 0.2 M NaCl, 5 mM CaCl2, 1 mM ZnCl). An overnight incubation in reaction buffer was performed. Next, a protein fixation step was performed by incubating the gel for 1 h at room temperature with a solution containing 40% (*v*/*v*) methanol and 10% (*v*/*v*) acetic acid. After two washes (10 min each) in bi-distilled water, staining was performed in colloidal Coomassie Brillant Blue G-250 (0.05%) dissolved in 1.6% (*v*/*v*) phosphoric acid, 8% (*w*/*v*) ammonium sulfate and 20% (*v*/*v*) methanol. After staining removal in 1% (*v*/*v*) acetic acid, gelatinase activities appeared as clear bands on a blue background. A digital scanner was used to capture zymography images.

### 4.7. Western Blot Analysis

SH-SY5Y cells (2 × 10^5^ cells/well) were grown in 6-well plate for 24 h. Cells were then treated with POE (7.2 μg/mL) for different time points, ranging from 1 h to 24 h in HI-FBS medium. After PBS washing, cellular lysis was performed in 80 μL of Laemmli buffer containing Tris-HCl (62.5 mM, pH 6.8), 10% (*w*/*v*) SDS, 25% (*w*/*v*) glycerol. Cellular lysates were centrifuged at 12,000× *g* for 1 min at 4 °C. BCA protein assay was used to determine the total protein concentration of each sample. Then, 25 μg proteins from each sample was mixed with 5% (*v*/*v*) β-mercaptoethanol and bromophenol blue, and heated at 95 °C for 5 min. Then protein samples were electrophoretically separated on 12% or 15% SDS-polyacrylamide gels and blotting onto PVDF membranes (0.45 μm).

Following a saturation step with a BSA blocking buffer [5% (*w*/*v*) BSA in 0.1% (*v*/*v*) PBS-Tween^®^-20], membranes were incubated overnight at 4 °C with primary antibodies opportunely diluted in blocking buffer. Primary antibodies used are listed in Table 3. Next, three washes in 0.1% (*v*/*v*) PBS-Tween^®^-20 solution were performed, then HRP-linked secondary antibodies of goat anti-rabbit IgG (1:10,000) or goat anti-mouse IgG (1:10,000) (Invitrogen, Waltham, MA, USA) were added to the membranes for 1h at room temperature. After the final wash three times in 0.5% (*v*/*v*) PBS-Tween^®^-20, Clarity Western ECL solution was used to detect protein bands using the Amersham^TM^ 600 Imager imaging system (GE Healthcare Life Science, Pittsburgh, PA, USA). Quantity One (version 4.6.6, Bio-Rad) was used as a tool for densitometric analysis of protein bands.

### 4.8. Cell Morphological Analysis

SH-SY5Y cells were seeded at a density of 2.5 × 10^4^ cells/well in 6-well plates with differentiating medium in the absence or presence of POE (7.2 μg/mL). Cells cultured in differentiating medium containing RA (10 μM) were used as a control of cell differentiation. After 7 h, 24 h and 5 days of incubation, cells were fixed in 2% (*v*/*v*) paraformaldehyde for 6 min at room temperature. Following PBS washing, cells were stained with Coomassie Brilliant Blue R-250. Excess dye was removed by repeated washes with bi-distilled water. Cell morphological changes were observed using a phase-contrast microscope. Cell body diameter of neurite-bearing cells was greater than twice the cell body diameter. The percentage of cells with neurites in a specific culture was calculated by counting at least 200 cells in each sample [47].

### 4.9. Immunofluorescence Staining

SH-SY5Y cells were seeded (15 × 10^3^ cells/well) on sterilized slides in a 24-well plate overnight. After appropriate cell treatments, mature neuron markers were investigated in SH-SY5Y cells. Regarding monosialotetrahexosylganglioside 1 (GM1) labeling, cells were incubated with CTX-B (Cholera toxin B-subunit) Alexa Fluor 488-conjugated (10 ng/mL) primary antibody and 33,342 Hoescht (10 μg/mL) for nuclei staining for 40 min at room temperature. Subsequently, cells were fixed in 2% (*v*/*v*) paraformaldehyde for 6 min and washed in PBS.

Regarding the other investigated markers (see Table 4) the fixation step was followed by a permeabilization step with a cold 1:1 acetone/ethanol solution for 4 min at room temperature. Cells were then incubated with a blocking solution (0.5% (*w*/*v*) BSA and 0.2% (*w*/*v*) gelatin in PBS) for 30 min at 37 °C and then with specific primary antibodies (listed in Table 4) for 1h at 37 °C, opportunely dilute in blocking solution. After 30 min of PBS washing, cells were incubated with Alexa 568-conjugated anti-rabbit secondary antibody (at 1:100 dilution in PBS). Finally, cells were washed twice with PBS and once with bi-distilled water to remove non-specifically bound antibodies. Fluorescent signals were detected using a Leica TCS SP8 confocal scanning microscope (Leica, Mannheim, Germany) equipped with a HeNe/Ar laser source for fluorescence measurements. Observations were made using a Leica HC PL Apo CS2 × 63 oil immersion objective. Images and signal fluorescence were composed and analyzed by Image J Fiji software. For signal analysis intensity integration has been used setting auto-threshold for each acquisition.

### 4.10. Statistical Analysis

Data are presented as the mean ± SD of triplicate from three independent experiments. Statistic was performed by one-way analysis of variance (ANOVA) followed by Tukey’s HSD test.

For Western blot analysis, difference between house-keeping-normalized intensity signals were assessed by Kruskall-Wallis test followed by Conover post-hoc test. Statistical differences were call at *p* < 0.05. ImageJ software (National Institutes of Health, Bethesda, MD, USA) was used to quantify fluorescence signals from immunostaining experiments.

## Figures and Tables

**Table 1:** Phenolic compounds with relative percentages identified in *P. oceanica* leaf extract (POE) by UPLC analysis [10].

Phenolic Compound	Chemical Structure	Composition (%)
D-(+)-Catechin	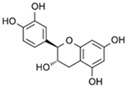	85
Ferulic acid	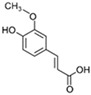	1.7
(−)-Epicatechin	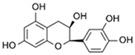	1.4
Chlorogenic acid	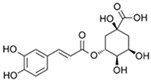	0.6
Gallic acid	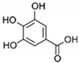	0.4
Others	–	11

**Table 2:** Biochemical properties of POE in terms of total polyphenols and carbohydrates content, and antioxidant and radical scavenging activity. Values (mg/mL) are expressed as mean ± SD from three independent colorimetric assays.

	TP	TC	Antioxidant	Radical Scavenging
Method	Folin-Ciocalteau	Phenol/Sulfuric acid	Ferrozine^®^	DPPH
Reference control	Gallic acid	Glucose	Ascorbic acid	Ascorbic acid
POE	3.2 ± 0.1	6.3 ± 1.4	1.2 ± 0.3	10.0 ± 2.0

**Table 3:** Details of primary antibodies used in Western blotting experiments.

Primary Antibody	Target	Dilution	Host	Source
SQTSM1/p62	SQTSM1/p62 protein	1:1000	Rabbit	Abcam
LC3	Microtubule-associated protein light chain 3	1:1000	Rabbit	Invitrogen
S6	Ribosomal protein S6	1:1000	Rabbit	Cell Signaling
p-S6	Ribosomal protein S6 (Ser235/236)	1:2000	Rabbit	Cell Signaling
α-Tubulin	α-Tubulin protein	1:1000	Mouse	Genetex

**Table 4:** Details of primary antibodies used in immunofluorescence staining experiments.

Primary Antibody	Target	Dilution	Host	Source
β3-Tubulin	total β3-tubulin protein	1:1000	Rabbit	Cell Signaling
GAP43	GAP43 protein	1:1000	Rabbit	Cell Signaling
NeuN	NeuN protein	1:1000	Rabbit	Cell Signaling
Synaptophysin	Synaptophysin protein	1:2000	Rabbit	Cell Signaling

## Data Availability

Not applicable.

## Supplementary Materials

The following are available online at https://www.mdpi.com/article/10.3390/md19100579/s1, Figure S1: Effect of 70% (*v*/*v*) EtOH vehicle in SH-SY5Y cells.
