# Supplementary material for: Posidonia oceanica (L.) Delile Dampens Cell Migration of Human Neuroblastoma Cells
Source: Mar Drugs. 2021 Oct 15;19(10):579. doi: 10.3390/md19100579 (PMC8539885; doi:10.3390/md19100579)

Figure S1. Effect of 70% (v/v) EtOH vehicle in SH-SY5Y cells. Representative images of Western blot analysis of the key autophagic markers levels investigated in vehicle-treated SH-SY5Y cells over time.

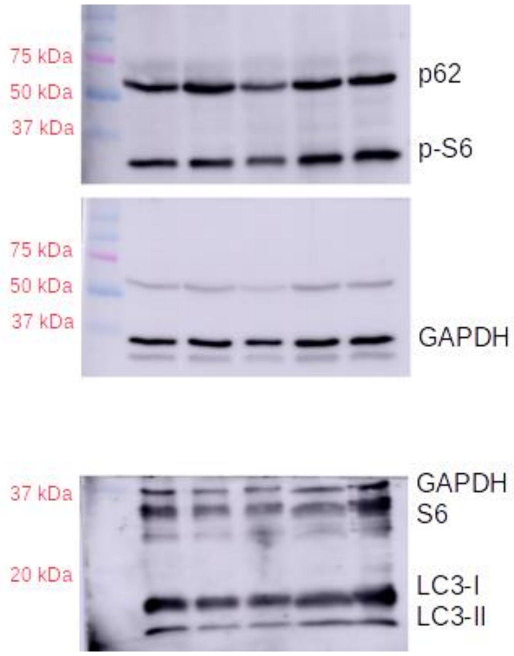

Supplement: Supplementary file 1 [file marinedrugs-19-00579-s001.zip › marinedrugs-1414559-supplementary.pdf]
